# Human cooperation in groups: variation begets variation

**DOI:** 10.1038/srep16144

**Published:** 2015-11-04

**Authors:** Pieter van den Berg, Lucas Molleman, Jaakko Junikka, Mikael Puurtinen, Franz J. Weissing

**Affiliations:** 1 Theoretical Research in Evolutionary Life Sciences (TRÊS), University of Groningen, 9747 AG Groningen, Netherlands; 2 The Centre for Decision Research and Experimental Economics (CeDEx), University of Nottingham, Sir Clive Granger Building, University Park, Nottingham NG7 2RD, UK; 3 University of Jyvaskyla, Department of Biological and Environmental Science, P.O.Box 35, FI-40014 University of Jyvaskyla, Finland; 4 University of Jyvaskyla, Centre of Excellence in Biological Interactions, P.O.Box 35, FI-40014 University of Jyvaskyla, Finland

## Abstract

Many experiments on human cooperation have revealed that individuals differ systematically in their tendency to cooperate with others. It has also been shown that individuals condition their behaviour on the overall cooperation level of their peers. Yet, little is known about how individuals respond to heterogeneity in cooperativeness in their neighbourhood. Here, we present an experimental study investigating whether and how people respond to heterogeneous behaviour in a public goods game. We find that a large majority of subjects does respond to heterogeneity in their group, but they respond in quite different ways. Most subjects contribute less to the public good when the contributions of their peers are more heterogeneous, but a substantial fraction of individuals consistently contributes more in this case. In addition, we find that individuals that respond positively to heterogeneity have a higher general cooperation tendency. The finding that social responsiveness occurs in different forms and is correlated with cooperativeness may have important implications for the outcome of cooperative interactions.

Scientists of various disciplines have since long been interested in cooperation^1^,^2^,^3^,^4^. For biologists, it is a major challenge to explain why natural selection sometimes favours behaviour that benefits other individuals (cooperation), especially when it is costly to perform^5^,^6^,^7^,^8^. The biological world is rife with examples of such behaviour (from birds and social insects to bacteria), and humans are no exception. In fact, human cooperation is in many ways more extreme than cooperation in most other animal species: we cooperate with non-related strangers and on enormous scales^9^,^10^,^11^. Not surprisingly, scholars from the social sciences also have a long tradition in studying cooperation^12^,^13^,^14^,^15^,^16^.

Studies using a range of methods have consistently shown that there is considerable individual variation in human cooperative behaviour. This is true for the general propensity to cooperate (cooperation tendency)^14^,^15^,^16^,^17^, but also for the ways people condition their cooperation on the cooperation of others (cooperation strategy)^18^,^19^,^20^,^21^. Importantly, the presence of those individual differences can significantly impact the outcomes of cooperative interactions in groups^19^,^22^. Recent theoretical studies have shown that the presence of even small amounts of variation in cooperative behaviour can be decisive for the evolution of cooperation^23^,^24^,^25^,^26^. Interestingly, also environmental variation in cooperation has been found to favour cooperative and forgiving strategies^27^,^28^,^29^,^30^. The success of cooperative and forgiving strategies in the presence of environmental variation stems from their ability to uphold profitable interactions even when partners mistakenly fail to cooperate, or when a cooperative act is mistakenly perceived as defection.

Given the prevalence of individual differences in cooperative behaviour, and the importance of variation for determining outcomes of cooperative interactions, it is surprising that little is known about how people condition their own cooperation on variation in cooperative behaviour in their social group. In studies designed to assess individuals’ cooperation strategies, response to heterogeneity is often disregarded. Many of these studies are based on the public goods game (PGG), where individuals are grouped and endowed with a sum of money, and then have to decide how much of the money to contribute to an account that benefits all members of their group. In this set-up, the total earnings of the group increase with increasing group member contributions, but individuals maximize their earnings by contributing nothing. To get an idea of the cooperation strategies employed by different individuals, subjects are asked how much they would contribute given various hypothetical average contribution levels of their fellow group members^18^,^31^. Such studies generally find that a large proportion of individuals is willing to contribute about equally much (or slightly less) as the average contribution of their fellow group members (they are often classified as ‘conditional cooperators’); others contribute nothing, regardless of the average peer contribution (‘free-riders’); still others contribute most when the average cooperation level of their interaction partners is intermediate.

One might expect that people take this variation in cooperation strategies into account when making decisions on their own degree of cooperation. In fact, some studies^32^,^33^,^34^ have reported that, on average, individuals tend to reduce their contribution to a public good if the contributions of their peers are more heterogeneous. However, it is not clear how this effect arises. Does the response to heterogeneity reflect a specific conditional strategy or a more general cautiousness in a variable environment? Do all individuals respond to heterogeneity in the same way, or are there consistent differences between individuals? If there are differences, how are they related to general cooperation tendency?

To answer these questions, we conducted an experiment that consisted of two parts. In the first part, subjects played ten rounds of a PGG in groups of four, where group composition changed in every round. In each round, subjects decided how to distribute an endowment of 20 points between their personal account and an account that benefitted all group members (see Methods for details). We interpret the average contribution of a subject to the group project in these ten rounds as a measure of the subject’s general ‘cooperation tendency’. In the second part, the same individuals decided how much they would contribute in a PGG, for ten hypothetical scenarios concerning the contributions of their fellow group members. In these scenarios, the hypothetical group member contributions were always either 0, 10 or 20 points, yielding a total of ten possible combinations of peer contributions. Six of these ten combinations were pairs of cases within which the average peer contribution was the same, but their heterogeneity was different. Comparing subjects’ conditional contributions between these scenarios allowed us to investigate how subjects respond to heterogeneity in peer contributions.

## Results

**Figure 1:**
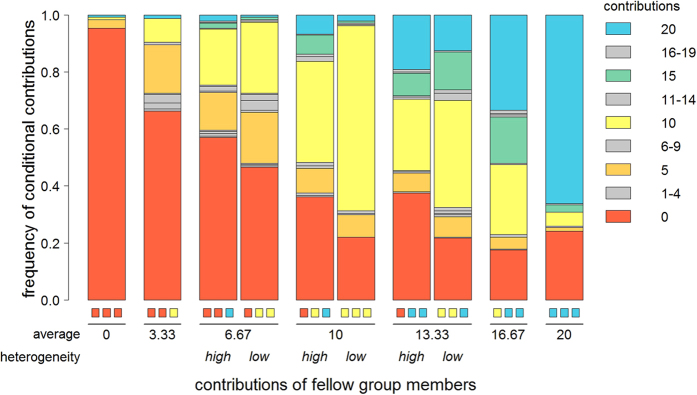
Contributions to the group project in response to various combinations of peer contributions. Each bar shows a breakdown of how subjects responded to a specific combination of contributions of the three other group members (indicated by the three coloured blocks under each bar). Bars are grouped together for cases that have the same average contribution of fellow group members, but where heterogeneity in peer contributions differs. Completely unresponsive individuals (contributing the same regardless of peer contributions) were omitted from the analysis (see Methods).

Figure 1 shows a detailed breakdown of the conditional contributions made in the second part of the experiment, for each combination of peer contributions. Overall, response contributions increased with peer contributions. If all fellow group members contributed nothing (leftmost bar), 95% of individuals also contributed nothing in response. Conversely, if all fellow group members contributed the maximum (rightmost bar), 72% of subjects also contributed the maximum in response. The grouped bars show pairs of scenarios where the average contribution of fellow group members is the same, but the heterogeneity in contributions differs. For example, the two middle bars (the 5^th^ and 6^th^ bar) show two cases where the average contribution is 10, but where the contributions are either heterogeneous (0, 10 and 20; bar 5) or homogeneous (three times 10; bar 6). From now on, we will focus on these pairs of scenarios. For all three pairs, Table 1 systematically compares the low- and the high-heterogeneity case concerning the average contribution of the subjects, the standard deviation of these contributions, and the frequency of extreme contributions (both minimum and maximum).

On average, individuals tended to contribute less when there was more heterogeneity in peer contributions (linear mixed model with subject as random factor, *P* <  0.001; see Supplementary Information, section 3 for a detailed overview of statistical methods). This is in accordance with earlier studies^32^,^33^,^34^. In addition, we observe that in two of the three comparisons the variation in response contributions was higher in case of more heterogeneity in peer contributions (for averages 10 and 13.33, Brown-Forsythe test: *P* < 0.001; for average 6.67, Brown-Forsythe test: *P* = 0.733). Finally, subjects were more likely to make extreme contributions when there was more heterogeneity in peer contributions – this was the case for both contributing the minimal amount 0 (logistic generalized mixed model with subject as a random factor, *P* = 0.001) and the maximal amount 20 (*P* < 0.001). Generally speaking, more heterogeneity in peer contributions caused subjects to make more extreme contributions themselves.

**Figure 2:**
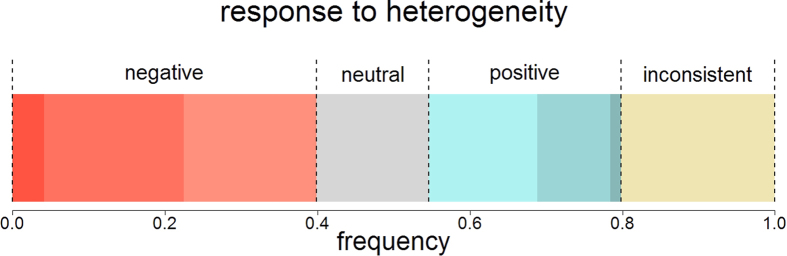
Response to heterogeneity in peer contributions. The bar shows a breakdown of subjects in how they responded to increased heterogeneity in peer contributions. We considered the three cases where the average of peer contributions was the same, but heterogeneity in peer contributions was different (grouped bars in Fig. 1). Subjects that contributed less when there was more heterogeneity in peer contributions in at least one of those cases, and never contributed more, were categorised as ‘negative’ responders to heterogeneity. Whether they contributed less in response to increasing heterogeneity in one, two, or all three cases is indicated with increasingly darker shading. ‘Positive’ responders to heterogeneity were classified similarly. If individuals contributed exactly the same for high and low heterogeneity in all three cases, they were classified as ‘neutral’ responders to heterogeneity. If individuals contributed less in some of the cases and more in others, they were classified as ‘inconsistent’.

Figure 2 reveals that individuals responded to heterogeneity in peer contributions in different ways. We classified subjects by comparing their contributions within each of the three pairs of scenarios that had the same average peer contribution, but different heterogeneity in peer contributions. If they contributed more in the cases with more heterogeneity, they were classified as ‘positive’ responders to heterogeneity, and if they contributed less, they were classified as ‘negative’ responders. If they contributed equally within all three comparisons, they were classified as ‘neutral’, and if they contributed more in case of high heterogeneity in some of the three comparisons, and less in others, they were classified as ‘inconsistent’. In line with the finding that, on average, contributions were lower in case of more heterogeneity (see Table 1), more subjects were classified as negative (39.9%) than positive (25.2%), but the fraction of positive individuals is substantial. Smaller fractions of individuals were neutral (14.7%) or inconsistent (20.2%) in their response to heterogeneity.

**Figure 3:**
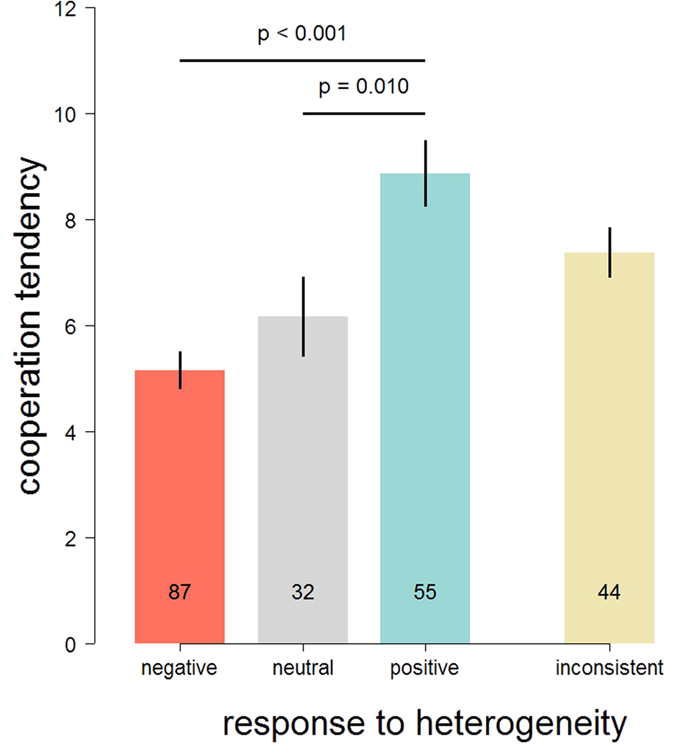
Response to heterogeneity in peer contributions is associated with cooperation tendency. Bars show the average and SEM of contributions over ten rounds of a public goods game, where group composition was randomised before every round, for negative, neutral, positive, and inconsistent responders to heterogeneity. Statistically significant differences between types are indicated (Tukey Honest Significant Differences), except for differences between inconsistent responders and any of the other groups. Numbers at the bottom of each bar indicate the number of subjects falling in this category.

Figure 3 shows that there is a clear relation between the response to heterogeneity as measured in the second part of the experiment, and the general tendency to cooperate as determined in the first part. Specifically, average contributions in the first part were 72% higher for individuals that responded positively to heterogeneity when compared to individuals that responded negatively; individuals that had a neutral or inconsistent response to heterogeneity were in between. This association cannot be explained by ‘spill-over’ effects^18^ between the two parts of the experiment; it is still observed when controlling for peer cooperation in the first part of the experiment (see Supplementary Information, section 3). Moreover, we still observe this clear difference when only considering the first interaction round of the first part, or the ‘unconditional contribution’ of the second part (see Methods) to determine general cooperation tendency. In these cases, contributions of individuals that positively responded to heterogeneity were respectively 50% and 40% higher than those of individuals that responded negatively; those differences were highly significant in both cases (See Supplementary Information, section 2 for graphic representations and details).

## Discussion

The results of our experiment can be summarised in three points. First, we confirm earlier observations that when the contributions of fellow group members to a public good are more heterogeneous, people on average respond by contributing less. However, this is not the whole story; more heterogeneity in peer contributions also leads to more variable (and more extreme) contributions in response (‘variation begets variation’). Second, we observe substantial individual differences in how people respond to the degree of heterogeneity in peer cooperation. Some individuals consistently contribute more when there is more heterogeneity, whereas others consistently contribute less. Smaller fractions were either neutral or inconsistent in their response to increased heterogeneity in peer cooperation. Third, we find a clear relation between general cooperation tendency and conditional responses to heterogeneity in peer contributions. Individuals that respond positively to heterogeneity in peer contributions tend to be more cooperative in a public goods game than individuals that respond negatively. Individuals that respond neutrally or inconsistently are intermediate in their cooperation tendency.

At first sight, it may seem that the classification of the individual variation that we made in our experiment (between positive, neutral, negative, and inconsistent individuals) does not reflect very clear differences between individuals. For example, an individual that was classified as ‘positive’ may in fact only have responded positively to heterogeneity in one of three comparisons, and neutrally in both others. Sure enough, our experiment should be considered as a first step in charting the individual differences in how people respond to heterogeneity in the cooperative behaviour of their peers; further studies will be needed to come to a more comprehensive account. Having said this, we observed that even individuals that responded marginally positively (the lightest blue shade in Fig. 2) to heterogeneity have a significantly higher cooperation tendency than individuals that responded marginally negatively (the lightest red shade in Fig. 2; see Supplementary Information, section 1 for details). The fact that even small differences in response to heterogeneity are associated with large differences in general cooperation tendency suggests that these differences cannot simply be regarded as random noise. In this study, we found an association between self-assessed competitiveness and response to variation; this link could be more thoroughly investigated (for instance, by measuring competitiveness experimentally rather than through self-assessment). Associations with other factors, such as aspects of personality, may also be interesting to explore.

Individual variation is currently attracting much attention in all the behavioural sciences, including biology^25^,^35^,^36^,^37^ (including cultural evolution research^21^,^38^,^39^), psychology and neuroscience^40^,^41^,^42^, and economics^18^,^31^,^43^. Biologists have shown that consistent individual differences in behavioural tendencies often have an adaptive explanation, and are likely to emerge in the course of evolution under a broad range of circumstances^37^,^44^. Moreover, various theoretical models^24^,^45^,^46^ show that the presence of consistent individual variation in social behaviour will induce the evolution of sensitivity and responsiveness to this variation. In line with the results reported here, these models predict that individuals differ consistently not only in their behaviour, but also in their response to the behaviour of others, and that both are correlated.

Our empirical results demonstrate that individuals vary not only in the degree of responsiveness, but also in the type of response to the social environment (i.e., there are positive and negative responders). This suggests that there exists a previously unrecognised dimension to social responsiveness. The observed link between the type of response and cooperation tendency can have important implications for the performance of cooperation strategies. For example, if cooperators typically assort together^47^,^48^,^49^, a positive response to heterogeneity may help in maintaining cooperation by ‘forgiving’ occasional non-cooperation by a member of the group due to mistakes or temporary inability^27^,^28^,^29^,^30^,^50^,^51^,^52^. The types of responsiveness we observe might be related to personality characteristics, such as differences in ‘lifestyle’. Theory predicts that evolution will often result in ‘pace of life’ syndromes, with individuals with a ‘fast’ and a ‘slow’ lifestyle coexisting in a population^44^,^53^. ‘Fast’ individuals are focused on short-term benefits, while ‘slow’ individuals are willing to take short-term losses if this is likely to result in longer-term benefits. One might speculate that cooperativeness and a positive response to variation are both facets of a slow lifestyle; ‘slow’ individuals are more cooperative, since they hope to elicit long-term cooperation, and they respond more positively to variation, since they interpret heterogeneity as an opportunity for longer-term cooperation rather than as a threat. Similar arguments may be used to interpret non-cooperativeness in a social dilemma and a negative response to variation as facets of a fast lifestyle. Formal evolutionary models have to be developed to check if these verbal arguments can be substantiated. Quite obviously, the implications of individual differences in type of responsiveness for the dynamics of social interactions and performance of cooperation strategies merit further empirical and theoretical scrutiny.

## Methods

A total of 240 subjects (71% female, mostly students) participated in experimental sessions consisting of 16 subjects each, at the University of Groningen (the Netherlands). Participation was by informed consent, and the experimental setup was approved by the Sociological Laboratory of the University of Groningen. The experimental sessions were carried out in accordance with the approved guidelines. During the sessions (lasting approximately one hour), subjects made a number of simultaneous and anonymous decisions on computers. Subjects earned points (50 points = €1) with the decisions they made, and were paid accordingly in cash at the end of the session (mean payoff: €14.87±1.90; ranging from €10.60 to €19.30; subjects were unaware of the earnings of others). Subjects received written instructions, which were also read out loud by the experimenters at the start of each session (see Supplementary Information, section 5 for instructions). Each session consisted of two parts that were separately explained on the computer screen before they started, after which subjects filled out a short quiz to check their comprehension. This experiment was conducted in conjunction with another experiment; see Supplementary Information, section 4 for details. The experiment was run with the experimental software z-Tree^54^ (code available upon request).

In the first part of the experiment, individuals played ten rounds of a PGG, in groups of four. Individuals were grouped randomly at the start of every round, and were made explicitly aware of this in the instructions before this part, as well as at the start of every new round. At the beginning of each round, subjects were allocated 20 points to distribute between a group project and their personal account. After all subjects had made their decision, the total contributions to the group project were doubled and divided equally among the group members (irrespective of their contributions), and subjects were shown their earnings (as well as the contributions and earnings of their fellow group members).

In the second part of the experiment, subjects were asked how much they would contribute (0–20 points) depending on the contributions of their fellow group members. We confronted them with ten hypothetical scenarios (on a single screen, in fixed order), where the contributions of their fellow group members were always 0, 10 or 20 points (see Fig. 1). Out of these ten scenarios, we pay particular attention to those pairs of cases that have the same average peer contribution, but differ in heterogeneity in peer contributions. Comparing subjects’ conditional contributions within these paired cases allowed us to determine how individuals respond to heterogeneity in peer contributions. In addition to the ten conditional contributions, each subject also entered one ‘unconditional contribution’ (where the choice was limited to 0, 10 or 20 points). This unconditional contribution was simply the contribution that individuals would make to the group project in case they did not know the contributions of their fellow group members. After this, one round of a PGG was played in randomly formed groups of four. From each group, three randomly chosen subjects automatically made their unconditional contribution, and the remaining subject made their corresponding conditional contribution. A total of 22 subjects (8.8%) contributed the same amount regardless of the peer contributions; all except one of these individuals were unconditional free-riders, contributing 0 for every scenario (the remaining individual was an unconditional cooperator, always contributing 20). Under our classification, these individuals would have been labelled as neutral responders to variation, but they are in fact completely unresponsive to peer contributions altogether. Therefore, these individuals were excluded from the analysis. Their exclusion did not affect the main results presented in this paper (see Supplementary Information, section 3).

## Additional Information

**How to cite this article**: Van den Berg *et al.* Human cooperation in groups: variation begets variation. *Sci. Rep.* **5**, 16144; doi: 10.1038/srep16144 (2015).

## Supplementary Material

Supplementary Information

## Figures and Tables

**Table 1:** Contribution to the group project in response to peer contributions differing in their mean and heterogeneity.

Peer contributions		Response contributions			
Mean	Heterogeneity	Mean	s.d.	% min	% max
**6.67**	*low*	4.32	4.61	41.7	0.5
	*high*	3.74	5.07	53.2	1.8
**10.00**	*low*	7.65	4.58	14.7	1.8
	*high*	6.90	6.20	30.3	6.9
**13.33**	*low*	9.42	6.32	14.2	13.3
	*high*	8.30	7.66	31.6	20.6

The table shows averages, standard deviations, and percentage of minimum and maximum contributions (respectively 0 and 20). In each row, two situations are compared where the peer contributions were equal on average, but differed in heterogeneity (see Fig. 1).
