# Supplementary material for: Human cooperation in groups: variation begets variation
Source: Sci Rep. 2015 Nov 4;5:16144. doi: 10.1038/srep16144 (PMC4632011; doi:10.1038/srep16144)
Supplement: Supplementary Information [file srep16144-s1.pdf]

SUPPLEMENTARY INFORMATION

Human cooperation in groups: variation begets variation

Van den Berg P, Molleman L, Junikka J, Puurtinen M & Weissing FJ

CONTENTS

1. Results for all subcategories of response to heterogeneity 2

2. Results using other measures of general cooperation tendency 3

3. Overview of statistics 4

4. Details of the experimental set-up 6

5. Experimental instructions 8

1. Results for all subcategories of response to heterogeneity

It may be argued that the classification of ‘response to heterogeneity’ in the main text of our experiment is somewhat crude. Even individuals that only contributed more in case of more heterogeneity in peer contributions in one of the three comparisons (and contributed equally in the other two cases) are classified as ‘positive responders’. Similarly, an individual that only contributed less in case of more heterogeneity in peer contributions in one case, was classified as ‘negative responders’. To investigate to what extent these seemingly small differences between weakly negative and weakly positive individuals are meaningful, we analysed the relationship between response to heterogeneity and cooperation tendency when considering all subcategories of individuals (as they are shown in Fig. 2 in the main text). Figure S1 shows that even the marginally positive and negative individuals have quite different cooperation tendencies.

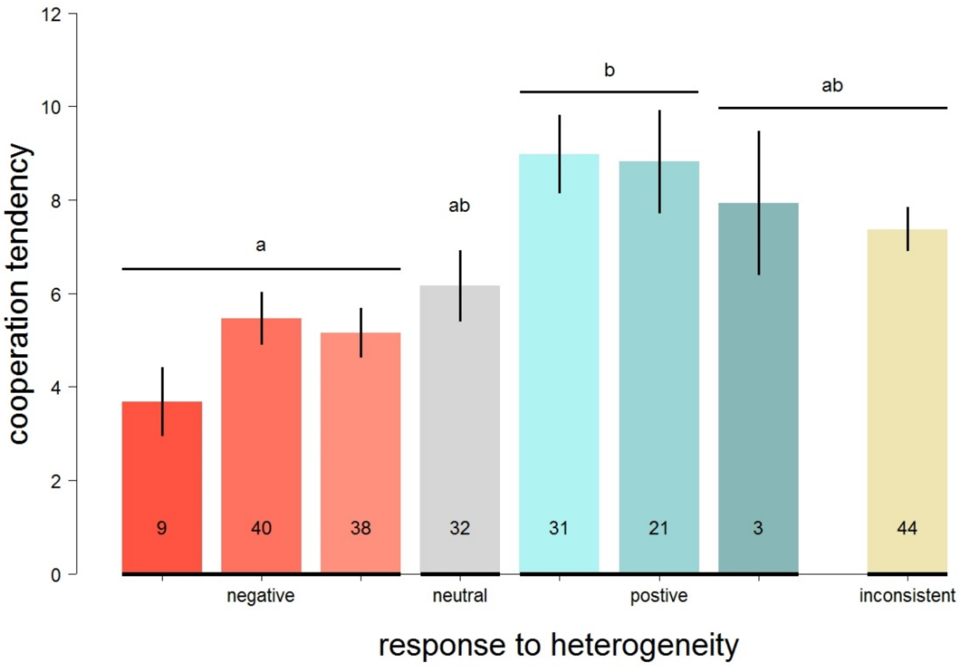

Figure S1. Response to heterogeneity in peer contributions is associated with cooperation tendency, even when the response to heterogeneity is weak. Bars show the average and SEM of contributions over ten rounds of a public goods game, where group composition was randomised before every round. The same data as in Fig. 3 of the main text are shown, but the ‘negative’ and ‘positive’ categories are further subdivided by degree of negativity or positivity, as in Fig. 2 of the main text. Negative responders to information are further subcategorised by whether they contributed less in response to increasing heterogeneity in one, two, or all three situations. Similar subcategories were made for positive responders to heterogeneity. The darker bars (furthest removed from the ‘neutral’ bar) show the most extreme responders, whereas the lighter bars (next to the ‘neutral’ bar) show the individuals that only responded positively or negatively to increased heterogeneity in one case, and responded neutrally in both other cases. Letters (**a** and **b**) indicate significant differences (Tukey HSD test); all bars indicated with **a** are significantly different from all bars indicated with **b**, whereas bars indicated with **ab** are not significantly different from either. Numbers at the bottom of each bar indicate the number of subjects falling in this category.

2. Results using other measures of general cooperation tendency

In our experiment, we measure general cooperation tendency by taking the average of subjects' contributions in ten consecutive one-shot rounds of a PGG. This is in principle a valid way to measure general cooperation tendency, because individuals are playing one-shot games; information from earlier rounds is not relevant when deciding how much to contribute. However, one may argue that the outcomes of earlier rounds may still have influenced subjects in their decisions. Therefore, we here check whether our results still hold when only considering the first interaction round (when individuals have no information whatsoever about the decisions of others). Figure S2*a* shows that this is indeed the case.

An alternative (and independent) measure of general cooperation tendency is the 'unconditional contribution' that individuals entered in the second part of the experiment. This unconditional contribution was used in the single round of PGG that was played in groups of four after the second part of the experiment. From each group, three randomly chosen subjects automatically contributed their unconditional contribution, and the remaining subject made their corresponding conditional contribution. Figure S2*b* shows that if this measure is used, the same pattern still emerges. This strongly suggests that the observed pattern is robust.

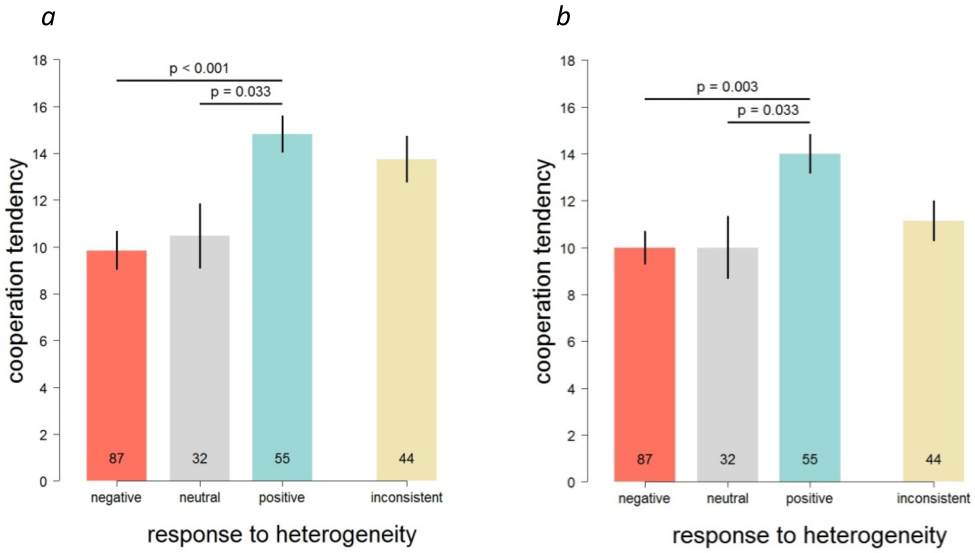

Figure S2. Response to heterogeneity in peer contributions is associated with cooperation tendency, also when using other measures of cooperation tendency. Bars show the average and SEM of contributions when considering (*a*) only the first PGG in the first part of the experiment, and (*b*) 'unconditional contributions' in the second part of the experiment. Statistically significant differences between types are indicated (Tukey HSD), except for differences between inconsistent responders and any of the other groups. Numbers at the bottom of each bar indicate the number of subjects falling in the respective category.

3. Overview of statistics

This section gives an overview of the statistical methods that were used in the study.

Factors affecting average contribution
We constructed a linear mixed model to determine which factors influence the response contribution levels when only considering the three pairs of cases that have equal average peer contribution, but different heterogeneity in contributions. We used a stepwise backwards elimination approach (Zuur *et al.*, 2009*), starting with a full model that contains as predictor variables the average peer contribution (the three levels are modelled as categorical factors), heterogeneity in peer contribution (including ‘high heterogeneity’ and ‘low heterogeneity’ as factors), and their interaction. Also, we included ‘individual’ as a random factor. The final model included both average peer contribution and heterogeneity in peer contribution as predictor variables, but not their interaction. We conclude that both average peer contribution and heterogeneity in peer contributions have a significant (respectively positive and negative) effect on response contributions. A summary of the final model is given below:

	Estimate	Std. error	t-value	P-value
(Intercept)	4.7951	0.3428	13.989	<0.001
heterogeneity in peer contributions	-0.9021	0.2348	-3.842	<0.001
average peer contribution (10)	3.5711	0.2876	12.418	<0.001
average peer contribution (13.33)	5.3142	0.2876	18.479	<0.001

In summary, average response contributions increase with average peer contributions, but decrease with increasing heterogeneity in peer contributions. This is consistent with earlier results.

Recent experiments indicate that the outcome of past social interactions in an experimental session may affect unrelated future cooperation decisions (Peysakhovich and Rand, 2015**). For our experiment, this may lead to the expectation that subjects who experienced higher average levels of cooperation in the first part of the experiment would contribute more to the public good in the second part. However, an extended regression analysis including peer cooperation levels in part 1 of the experiment reveal that they had no significant effect on public good contributions in part 2. There was a small yet significantly positive effect of the average cooperation levels in the stable groups (rounds 11-25; see Section 4 of this Supplementary Information), but including this factor in the regression analysis did not alter our main conclusions; both average peer contribution and heterogeneity in peer contributions have on average a significant (respectively positive and negative) effect on response contributions.

*Zuur A, Ieno EN, Walker N, Saveliev AA, Smith GM. 2009. Mixed Effects Models and Extensions in Ecology with R. Springer (New York).
** Peyshakhovich A, Rand D. 2015 Habits of virtue: creating norms of cooperation and defection in the laboratory. *Management Science* (forthcoming)

Factors affecting the incidence of extreme contributions
To determine which factors influence the incidence of extreme contributions we constructed a mixed-effects logistic regression model both for the incidence of contributions of 0 and the incidence of contributions of 20. We used a stepwise modelling approach, as described for the linear mixed model above, and included the same predictor variables in the first step. The final model for **contributions of 0** contained average peer contribution, heterogeneity in peer contributions, and their interaction as predictors:

	Estimate	Std. Error	z-value	P-value
(Intercept)	-0.6805	0.2617	-2.600	0.009
heterogeneity in peer contributions	0.8905	0.2718	3.276	0.001
average peer contribution (10)	-2.5345	0.3317	-7.642	<0.001
average peer contribution (13.33)	-2.5948	0.3346	-7.754	<0.001
average (10) * heterogeneity	0.7127	0.4164	1.712	0.087
average (13.33) * heterogeneity	0.8907	0.4179	2.131	0.033

The final model **for contributions of 20** contained average peer contribution and heterogeneity in peer contributions as predictors:

	Estimate	Std. Error	z-value	P-value
(Intercept)	-14.3402	1.5627	-9.177	<0.001
heterogeneity in peer contributions	1.9519	0.4378	4.458	<0.001
average peer contribution (10)	2.5724	0.7352	3.499	<0.001
average peer contribution (13.33)	6.4348	0.8979	7.166	<0.001

In summary, heterogeneity in peer contributions affects the incidence of extreme contributions in both ways; it has a positive effect on both the frequency of contributions of 0 and the frequency of contributions of 20. Average contribution also had an effect in both cases, but in opposite directions.

Effect of exclusion of unresponsive individuals

For the results presented in this study, individuals that were completely unresponsive (always made the same contribution, regardless of peer contributions) were excluded from the analysis. These were in total 22 subjects (8.8%); 21 unconditional free-riders (always contributing 0), and one unconditional cooperator (always contributing 20). The exclusion of these individuals from our analysis does not affect our conclusions. Their inclusion would increase the frequency of response contributions of 0 and 20 with the same amount for each combination of peer contributions (resulting in the same amount of extra red and blue in each bar of Fig. 1). Since all unresponsive individuals would have been classified as ‘neutral responders to heterogeneity’ (resulting in a larger ‘neutral’ group in Fig. 2), their exclusion does not affect the differences in cooperation tendency that we observe between positive and negative responders to heterogeneity (see Fig. S3).

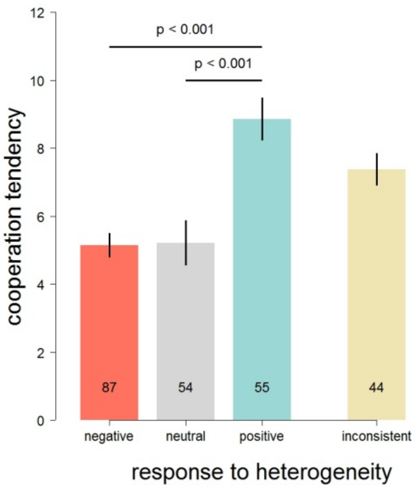

Figure S3. Response to heterogeneity in peer contributions is associated with cooperation tendency, also when including unresponsive individuals in the analysis. Bars show the average and SEM of contributions. Statistically significant differences between types are indicated (Tukey HSD), except for differences between inconsistent responders and any of the other groups. Numbers at the bottom of each bar indicate the number of subjects falling in the respective category.

4. Details of the experimental set-up

The current experiment was part of a larger experiment that was designed to test the effects of positive assortment on cooperation in human groups. Here, we give a full description of the entire experimental set-up.

At the start of each session, all subjects received written instructions that were also read aloud by one of the experimenters (full instructions are included in chapter 4 of the Supplementary Materials). Individuals did not know that the experiment was designed to test the effects of assortment or information about assortment in the first rounds of the experiment, or that the first rounds were used to measure general cooperation tendency for the current study.

As described in the main text, subjects first anonymously interacted in a Public Goods Game (PGG) for 10 rounds in groups of four, with changing group compositions in each round. The average contribution in these ten rounds was used as a measure of individual cooperation tendency in this study.

After the first 10 rounds, subjects played another 15 rounds of the PGG. This part was designed to test the effects of assortment and information about assortment. Each session was assigned to one of three treatments: *i)* assortment with information, *ii)* assortment without information, and *iii)* no assortment. Prior to this part, subjects received new instructions relevant to the treatment of their session. In the assortment treatments (*i* and *ii*), subjects were assorted in groups of four based on their decisions in the first 10 rounds of the PGG; the individuals that contributed most were grouped together, as were the individuals that contributed least, and the individuals that were in between. Individuals then played 15 more rounds of the PGG in these assorted groups, with fixed group membership over all rounds. In treatment *i*, individuals were made aware of the assortment regime, and were told in which group they were to be assorted (*i.e.*, they had information about the general cooperation tendency of their fellow group members). In treatment *ii*, individuals did not have this information, and were only told that

they would now interact in fixed groups. In treatment *iii*, individuals were grouped randomly, and were only told that they would now interact in fixed groups.

After these 15 rounds, the second part of the current study ensued: individuals were asked how much they would contribute in various situations with different contributions of fellow groups members (as explained in the main text).

To demonstrate that the results described in the main text of this study were not affected by the assortment regimes described above, we show our results separately for each treatment in Fig. S3 bel[illegible]

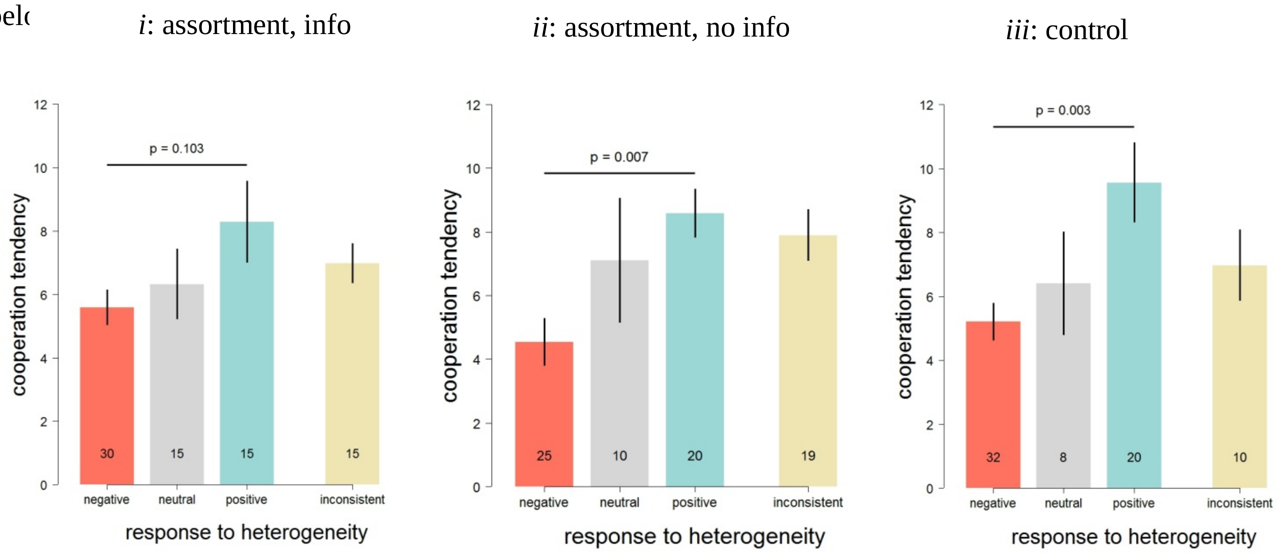

Figure S4. Response to heterogeneity in peer contributions is associated with cooperation tendency independent of experimental treatment. Bars show the average and SEM of contributions over 10 rounds of a public goods game, where group composition was randomised before every round, separately for individuals who had earlier experienced treatments *i* (assortment with information), *ii* (assortment without information) and *iii* (control; no assortment). All three graphs show the *P*-value of a Tukey HSD test, comparing the cooperation tendency of negative and positive individuals. Only for the subset of individuals that were in treatment *i*, response to heterogeneity does not significantly predict general cooperation tendency, but even in this case the effect is in the same direction as the overall pattern. Numbers at the bottom of each bar indicate the number of subjects falling in the respective category.

5. Experimental instructions

Below, the experimental instructions are shown that subjects received at the start of the session. Those instructions were read aloud by one of the experimenters.

Introduction

Welcome to this experiment!

This session will last for approximately one hour. During the session it is **not allowed to talk** or communicate with the other participants. If you have a question, please raise your hand and one of us will come to you to answer it.

In this experiment you will play a game in which you can earn points. After the experiment, these points will be converted into real money (50 points = 1€). The amount you earn depends on your decisions and the decisions of others. At the end of the experiment, you are asked to fill out a Questionnaire.

The money you earn will be paid to you in cash individually in the reception room. **Please stay seated at the end of the session** until your desk number is called. We will not inform any of the other participants about your earnings.

Instructions

In this experiment you will play a game. The game is subdivided in rounds. First, you will play a block of **10 rounds**. At the beginning of each round, the participants in the room are randomly divided into four groups of four players. Group members are anonymous, so you will not know who the other members are, and the other members will not know who you are. The **groups are randomly formed in the beginning of each new round**. This means that you are in a different group in every round. After the first block of 10 rounds, you will receive new instructions on the computer screen for the rest of the experiment.

<u>Progress of the game:</u>

1. At the start of a round, you are given 20 points.
2. Next, you decide how many points (0-20) you contribute to a group project, and how many points you keep for yourself. At the same time, the other three members of your group make their decision about the use of their points.
3. After all group members have made their decision, all points contributed to the group project are summed, and the game organizer doubles this number of points.
4. The doubled number of points is divided equally among the group members (irrespective of how much they contributed to the group project).
5. The points you earn in a round will be stored in the computer memory. These points cannot be used in following rounds. At the end of each round, you are informed about the contributions and earnings in your group. When a new round begins, new groups are formed and you are given another 20 points to start with.
6. After ten rounds, the first block of rounds is over, and new instructions will be given on the computer screen.

Your points after a round:

The points you keep for yourself
plus
the points you earn from the group project

Group project – Example 1

All 4 players contribute 20 points to the group project: 80 points in total.

The number of points in the group project is doubled to 160 (2x80) points.

The points from the group project are divided equally among the four players: 40 points for each.

At the end of the round, each player has earned 40 points.

Group project – Example 2

Three players (A, B and C) contribute 20 points to the group project; one player (D) contributes 0 points. Hence, in total 60 points are contributed to the group project.

The number of points in the group project is doubled to 120 (2×60) points.

The points from the group project are divided equally among the four players; 30 points for each.

In this round, players A, B and C obtain 30 points (zero points kept for themselves *plus* 30 points earned from the group project), and player D obtains 50 points (20 points D kept for himself *plus* 30 points from the group project).
